# Supplementary material for: The interactome of intact mitochondria by cross-linking mass spectrometry provides evidence for coexisting respiratory supercomplexes
Source: Mol Cell Proteomics. 2017 Dec 8;17(2):216–32. doi: 10.1074/mcp.RA117.000470 (PMC5795388; doi:10.1074/mcp.RA117.000470)
Supplement: Supplemental Data [file supp_RA117.000470_133922_0_supp_23978_fzffwf.zip › spectra_annotation/mito_DR_spectra_annotation/394-1-1-1-5-1.pdf]

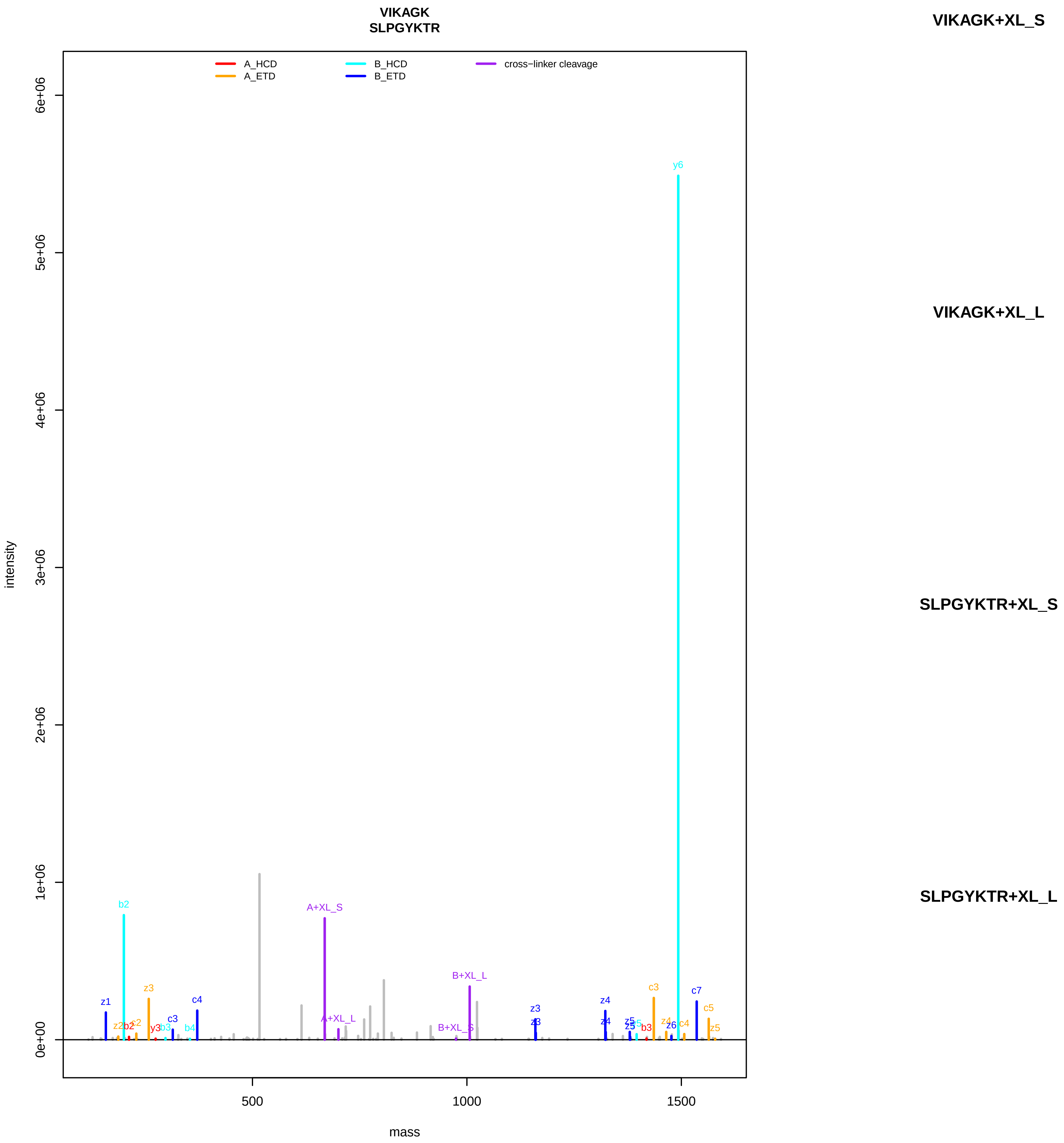

VIKAGK
SLPGYKTR
A_HCD
A_ETD
B_HCD
B_ETD
cross−linker cleavage
intensity
mass
0e+00
1e+06
2e+06
3e+06
4e+06
5e+06
6e+06
500
1000
1500
y6
b2
A+XL_S
B+XL_L
A+XL_L
B+XL_S
z1
z2
b2
c2
z3
y3
c3
b3
b4
c4
z3
z3
z4
z4
z5
z5
y5
b3
c3
z4
z6
c4
c7
c5
z5
VIKAGK+XL_S
VIKAGK+XL_L
SLPGYKTR+XL_S
SLPGYKTR+XL_L
